# Association of autoimmune diseases with the occurrence of osteoarthritis: a gene expression and Mendelian randomization study

**DOI:** 10.3389/fmed.2024.1435312

**Published:** 2024-09-05

**Authors:** Jing Dan, Huai Min Lu, Xun Zhou, Hong Yuan Wang, Jia Hao Wang

**Affiliations:** Department of Sports Medicine and Rehabilitation, Affiliated Sport Hospital of CDSU, Chengdu, China

**Keywords:** osteoarthritis (OA), autoimmune diseases, Mendelian randomization (MR), genome-wide association study (GWAS), metabolism-related

## Abstract

**Background:**

Observational studies have indicated a potential association between autoimmune diseases and the occurrence of Osteoarthritis (OA), with an increased risk of mortality among affected patients. However, whether a causal relationship exists between the two remains unknown.

**Methods:**

In the Mendelian randomization (MR) study, we accessed exposure Genome-wide association study (GWAS) data from both the MRC Integrative Epidemiology Unit (MRC-IEU) and the FinnGen consortium. GWAS data for OA were obtained from MRC-IEU. We employed univariable, multivariable, and reverse MR analyses to explore potential associations between autoimmune disorders and OA. Additionally, a two-step mediation MR analysis was performed to investigate indirect factors possibly influencing the relationship between autoimmune disorders and OA. Afterward, we conducted an observational analysis to further explore the relationship between autoimmune disease and occurrence as well as of OA using a real-world database (the MIMIC-IV database). Based on public gene expression sequencing data, we further explored the potential shared pathogenesis between autoimmune diseases and OA.

**Results:**

In our univariable MR study, we identified five autoimmune diseases that are associated with OA. These include Celiac disease (OR = 1.061, 95% CI = 1.018–1.105, *p* = 0.005), Crohn’s disease (OR = 1.235, 95% CI = 1.149–1.327, *p* = 9.44E-09), Ankylosing spondylitis (OR = 2.63, 95% CI = 1.21–5.717, *p* = 0.015), RA (OR = 1.082, 95% CI = 1.034–1.133, *p* = 0.001), and Ulcerative colitis (OR = 1.175, 95% CI = 1.068–1.294, *p* = 0.001). In the mediation effect analysis, it was found that there is no correlation between cytokines and autoimmune diseases and OA. Based on transcriptome data analysis, it was found that metabolism-related pathways play a key role in the co-morbidity of autoimmune diseases and OA.

**Conclusion:**

Our findings revealed that genes associated with Celiac disease, Crohn’s disease, Ankylosing spondylitis, RA, and Ulcerative colitis were independently linked to the development of OA. Furthermore, we conducted an analysis of potential pathogenic genes between these diseases and OA, offering a novel approach for the simultaneous treatment of multiple conditions.

## Introduction

Osteoarthritis (OA) is the most common form of arthritis, characterized by progressive degeneration of the articular cartilage and subchondral bone, leading to joint pain and disability (1, 2). It is a major cause of morbidity and disability worldwide, affecting millions of people, especially the elderly. The prevalence of OA increases with age, with a higher prevalence among women compared to men. It is estimated that approximately 10% of men and 18% of women over the age of 60 have symptomatic OA. The risk factors for OA include age, gender, obesity, joint injury, and genetics. Age is the most significant risk factor for OA, with the prevalence increasing sharply after the age of 50. Women are more likely to develop OA, especially after menopause, due to hormonal changes. Estrogen is crucial for regulating the metabolic equilibrium of joint cartilage. As ovarian function declines and estrogen levels decrease significantly after menopause, there is a reduction in the production of collagen fibers and egg polysaccharides by chondrocytes, leading to a decline in cartilage matrix. This ultimately results in the gradual thinning, softening, and breakdown of cartilage, ultimately leading to the development of osteoarthritis. Obesity is also a major risk factor for OA, particularly in weight-bearing joints such as the knees and hips (3, 4). Joint injuries, such as fractures or ligament tears, can also increase the risk of developing OA in the affected joint. Genetics play a role in the development of OA, with certain gene variants associated with an increased risk of the disease.

Autoimmune diseases are a group of disorders in which the immune system mistakenly attacks the body’s own tissues, leading to inflammation and tissue damage. Examples of autoimmune diseases include rheumatoid arthritis, systemic lupus erythematosus, and psoriatic arthritis (5). While autoimmune diseases primarily affect the joints, they can also have systemic effects on other organs and tissues. The relationship between autoimmune diseases and osteoarthritis is complex and not fully understood. While osteoarthritis is traditionally considered a non-inflammatory condition, recent studies have suggested that inflammation may play a role in the pathogenesis of the disease. Inflammatory cytokines, such as tumor necrosis factor-alpha (TNF-α) and interleukin-1 (IL-1), have been found to be elevated in the synovial fluid of OA patients, indicating a potential inflammatory component in the disease process (6, 7).

Several studies have investigated the association between autoimmune diseases and osteoarthritis. A study by Liu et al. found that patients with rheumatoid arthritis were at an increased risk of developing knee osteoarthritis compared to the general population (8, 9). Another study by Johnson et al. reported a higher prevalence of osteoarthritis in patients with systemic lupus erythematosus compared to healthy controls (10, 11).While there have been studies examining the potential link between autoimmune diseases and osteoarthritis, the current research primarily focuses on causal analysis and does not necessarily establish a direct correlation between the two. Furthermore, there is a lack of research on the relationship between all autoimmune diseases. As a result, this study utilized Mendelian randomization to investigate the potential correlation between autoimmune diseases and osteoarthritis. Additionally, gene expression correlation analysis was conducted on relevant diseases in order to explore the potential connection between the genes involved in both conditions.

## Materials and methods

### Data sources

#### Study population and data sources

Our analysis included a total of 11 different autoimmune diseases, which were obtained from the FinnGen consortium (R10) and the MRC-IEU online database (Crohn’s disease), Multiple sclerosis, Celiac disease, Psoriasis, Ankylosing spondylitis, Rheumatoid arthritis (RA), Type 1 diabetes (T1DM), Ulcerative colitis, Primary biliary cholangitis (PBC), primary sclerosing cholangitis, Systemic lupus erythematosus(SLE). These diseases were: Systemic lupus erythematosus, Rheumatoid arthritis, Crohn’s disease, Ulcerative colitis, Type 1 diabetes, Celiac disease, Psoriasis, Ankylosing spondylitis, Multiple sclerosis, Primary biliary cholangitis (PBC), and primary sclerosing cholangitis.

#### Mediator factors

In our study, we selected 15 mediator factors that may be affected by autoimmune diseases, including levels of immunoglobulins and serum cytokines. These factors included IgA, IgM, IgG, IgE for immunoglobulin levels, and IL-1α, IL-6, IL-12, IL-4, IL-10, IL-13, TGF-β1, TGF-α2, TGF-γ3, TNF-α, and IFN-γ for cytokines. Each genetic tool was considered significant at a genome-wide level (*p <* 5 × 10–6) and independent from each other (LD r2 < 5,000 kb within 0.01).

#### Outcome and transcriptome dataset

Our study included data from the FinnGen consortium (R10) on Osteoarthritis, with a sample size of 379,661 Europeans (6,991 cases and 372,620 controls) and 21,311,942 SNPs. Additionally, we obtained data from the GEO database (GSE55235, GSE41038, GSE55235, GSE95095, GSE48958, GSE112102) for all autoimmune diseases causally related to Osteoarthritis and Osteoarthritis itself. More detailed information on these datasets can be found in Supplementary Table S1.

#### Other factors and Mendelian randomization

To fulfill the second condition of Mendelian randomization, we thoroughly searched the PhenoScanner database for established relationships between instrumental SNPs and potential confounding variables. As the most influential genetic loci associated with autoimmune diseases are often found within the major histocompatibility complex (MHC), which is closely linked to adaptive immune responses, we took careful steps to exclude variants within this region. Specifically, we defined the exclusion region as base positions 24,000,000 to 35,000,000 on chromosome 6 (GRCh37) to mitigate potential impacts from autoimmune diseases and the complex LD structure within the MHC region.

### Instrumental variable selection

To validate the hypothesis of two-sample MR, we utilized independent single nucleotide polymorphisms (SNPs) that showed a strong association with the exposure and reached genome-wide significance (*p <* 5. × 10–6). These SNPs were carefully selected to ensure minimal linkage disequilibrium (r2 < 0.01) and maintain their independence. However, for the reverse MR analysis, there were not enough SNPs that met the same inclusion criteria, prompting us to adopt the following standards: *p* < 1. × 10–5, r2 < 0.001 within 10,000 kb. The strength of our instrumental variables was assessed using the F-statistic (beta2/se2), with a value greater than 10 indicating robust instruments. After removing palindromic SNPs, the remaining selected SNPs were used as instrumental variables for subsequent analysis.

#### Mendelian randomization analysis

The “TwoSampleMR” package was utilized for MR analysis. For proteins with only one instrumental variable, the log odds ratio change in risk of ankylosing spondylitis per standard deviation (SD) increase represented by the instrumental variable for the autoimmune disease was estimated using the Wald ratio. The Inverse Variance Weighted (IVW) method was employed to obtain MR effect estimates for autoimmune diseases with multiple instrumental variables. Additional analyses were conducted, including simple mode, weighted mode, weighted median, and MR Egger, to account for horizontal pleiotropy. MR Egger results were utilized only if the intercept indicated the presence of horizontal pleiotropy. BH Corrected *p*-values were used, with a significance level of *p* < 0.05. Heterogeneity was assessed using Cochrane’s Q statistic. Horizontal pleiotropy was evaluated using the MR-Egger intercept and MR-PRESSO methods. In case of outlier detection, they were removed and MR causal estimates were re-evaluated. Results from MR PRESSO correction were also included in the main results as they utilized the IVW method. Multivariable Mendelian randomization studies were conducted to adjust for potential confounders related to the outcomes of interest. Additionally, multivariable IVW-MR analysis was performed to adjust for Systemiclupus erythematosus in the model. In multivariable MR (MVMR) analysis, the IVW model was the primary method, with the MR-Egger method as a supplementary approach.

Random-effects models were used to combine MR results obtained from the FinnGen and MRC-IEU databases. All statistical analyses were carried out using the Mendelian Randomization (0.4.2), TwoSampleMR (0.5.7), MR-PRESSO (1.0), MVMR (0.3), and meta (4.11.0) packages in R version 4.2.2.

### Differential expression and enrichment analysis

We conducted a differential analysis on all autoimmune diseases linked to ankylosing spondylitis, including ankylosing spondylitis itself. The overlapping diseases were then subjected to differential gene enrichment analysis in order to investigate the specific functions of the genes involved. Our data analysis and visualization were performed using R software version 4.2.2, with measurement data reported as mean and standard deviation. The limma package was utilized for differential expression analysis of sequencing data. For comparisons between two or more groups, a *t*-test was used for normally distributed data, while non-parametric tests were used for non-normally distributed data. The threshold for differential analysis was set at |logFC| > 0.7 and a *p*-value <0.05. To gain a deeper understanding of gene functions, we utilized Gene Ontology (GO) and Kyoto Encyclopedia of Genes and Genomes (KEGG) datasets in our analysis. The ClusterProfiler tool was employed to explore the complex signaling pathways and functions associated with these genes.

## Results

### Mendelian randomization

**Figure 1:**
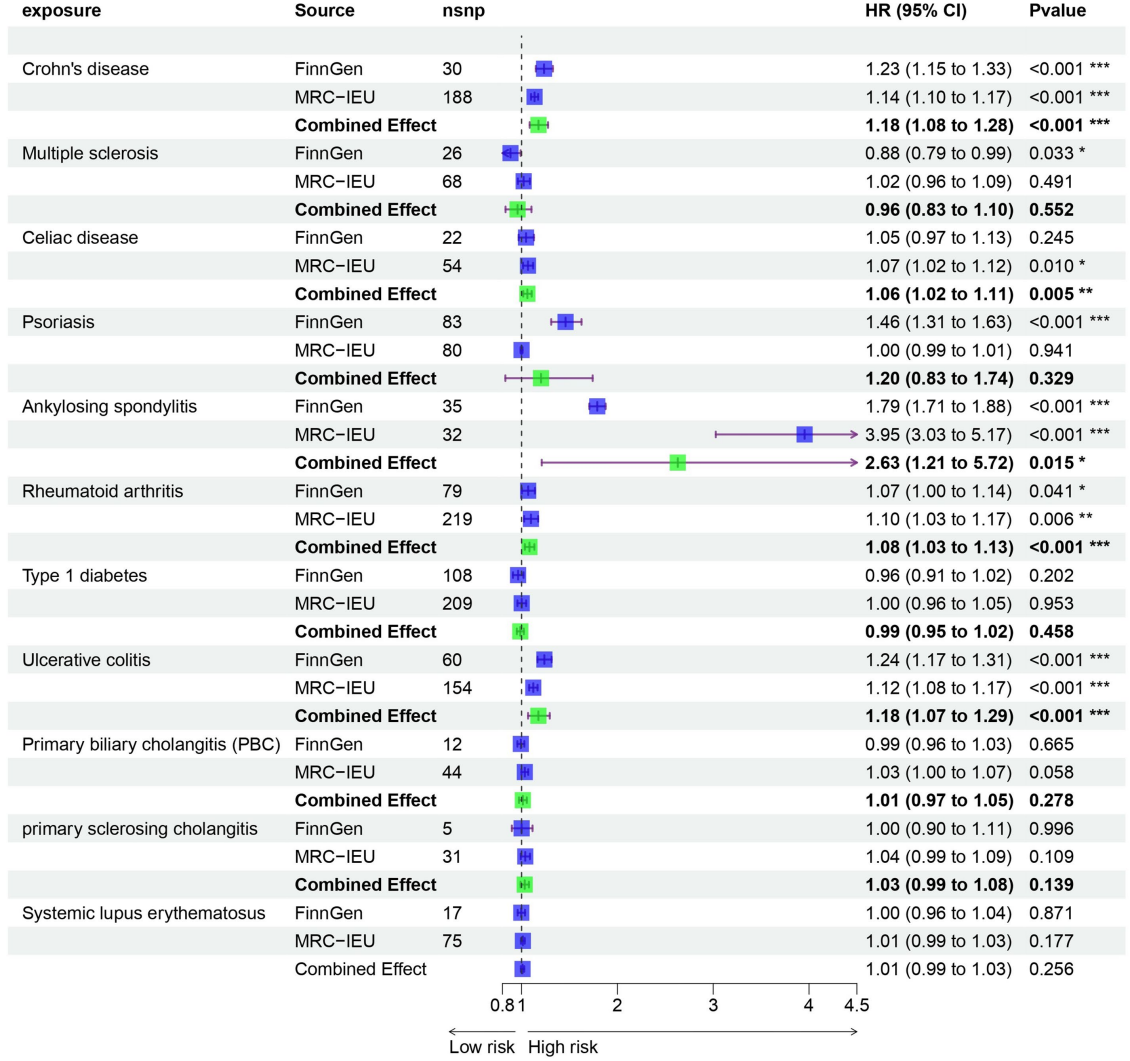
Univariate Mendelian randomization analysis of immune diseases and osteoarthritis.

Prior to performing Mendelian randomization, a sample overlap analysis was conducted for the included samples. This analysis identified a level of sample overlap between patients with osteoarthritis and other samples (see Supplementary Table S1). In our univariable MR study, we identified five autoimmune diseases that are associated with OA. These include Celiac disease (OR = 1.061, 95% CI = 1.018–1.105, *p* = 0.005), Crohn’s disease (OR = 1.235, 95% CI = 1.149–1.327, *p* = 9.44E-09), Ankylosing spondylitis (OR = 2.63, 95% CI = 1.21–5.717, *p* = 0.015), RA (OR = 1.082, 95% CI = 1.034–1.133, *p* = 0.001), and Ulcerative colitis (OR = 1.175, 95% CI = 1.068–1.294, *p* = 0.001). These five autoimmune diseases are all risk factors for the development of OA (Figure 1). Notably, the strongest association was observed between ankylosing spondylitis and OA, with an OR of 2.63. Our analysis utilized data from two datasets to provide a comprehensive evaluation. In addition to the diseases that were significant in both datasets, we also observed differences in the association of Psoriasis with OA in the FinnGen dataset compared to the MRC-IEU dataset. Furthermore, Multiple sclerosis was found to be a protective factor in the FinnGen dataset, while it was a risk factor in the MRC-IEU dataset (Table 1).

**Table 1:** The impact of immune diseases on osteoarthritis.

Exposure	OR	LCI	UCI	*P*-value	Heterogeneity_test
Crohn’s disease	1.176372	1.084645	1.275856	8.79E-05	0.037702
Multiple sclerosis	0.958172	0.832185	1.103231	0.552471	0.027483
Psoriasis	1.202815	0.830456	1.742132	0.328554	9.05E-12
Ankylosing spondylitis	2.630371	1.2102	5.717115	0.014621	1.05E-08
Ulcerative colitis	1.175381	1.067774	1.293832	0.000972	0.006926
Celiac disease	1.06062	1.017742	1.105305	0.005187	0.679599
Rheumatoid arthritis	1.082439	1.033719	1.133454	0.000748	0.570561
Type 1 diabetes	0.986366	0.95065	1.023424	0.457751	0.298879
Primary biliary cholangitis (PBC)	1.013009	0.97324	1.054403	0.278014	0.107006
Primary sclerosing cholangitis	1.032391	0.989653	1.076974	0.139467	0.532392
Systemic lupus erythematosus	1.010004	0.992789	1.027518	0.256441	0.453698

**Figure 2:**
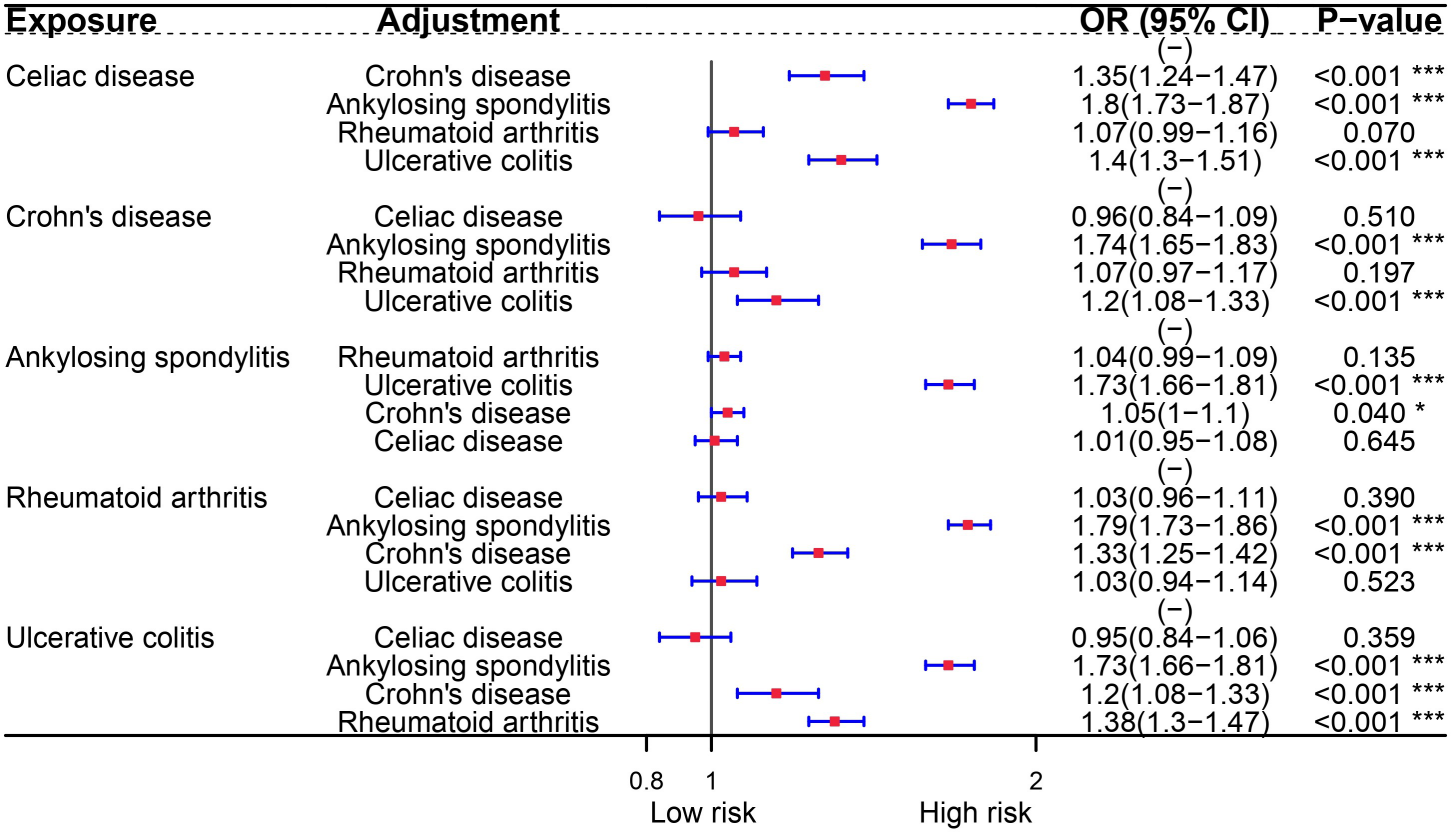
Multivariate Mendelian randomization analysis of immune diseases and osteoarthritis.

In the multivariable analysis of the FinnGen consortium dataset, we examined the potential association between autoimmune diseases and OA. Surprisingly, our findings indicate that only Celiac disease is an independent risk factor for OA. This association persists even after adjusting for other risk diseases. Furthermore, our analysis reveals that RA and Crohn’s disease remain associated with OA, even after controlling for ankylosing spondylitis and ulcerative colitis (Figure 2).

**Figure 3:**
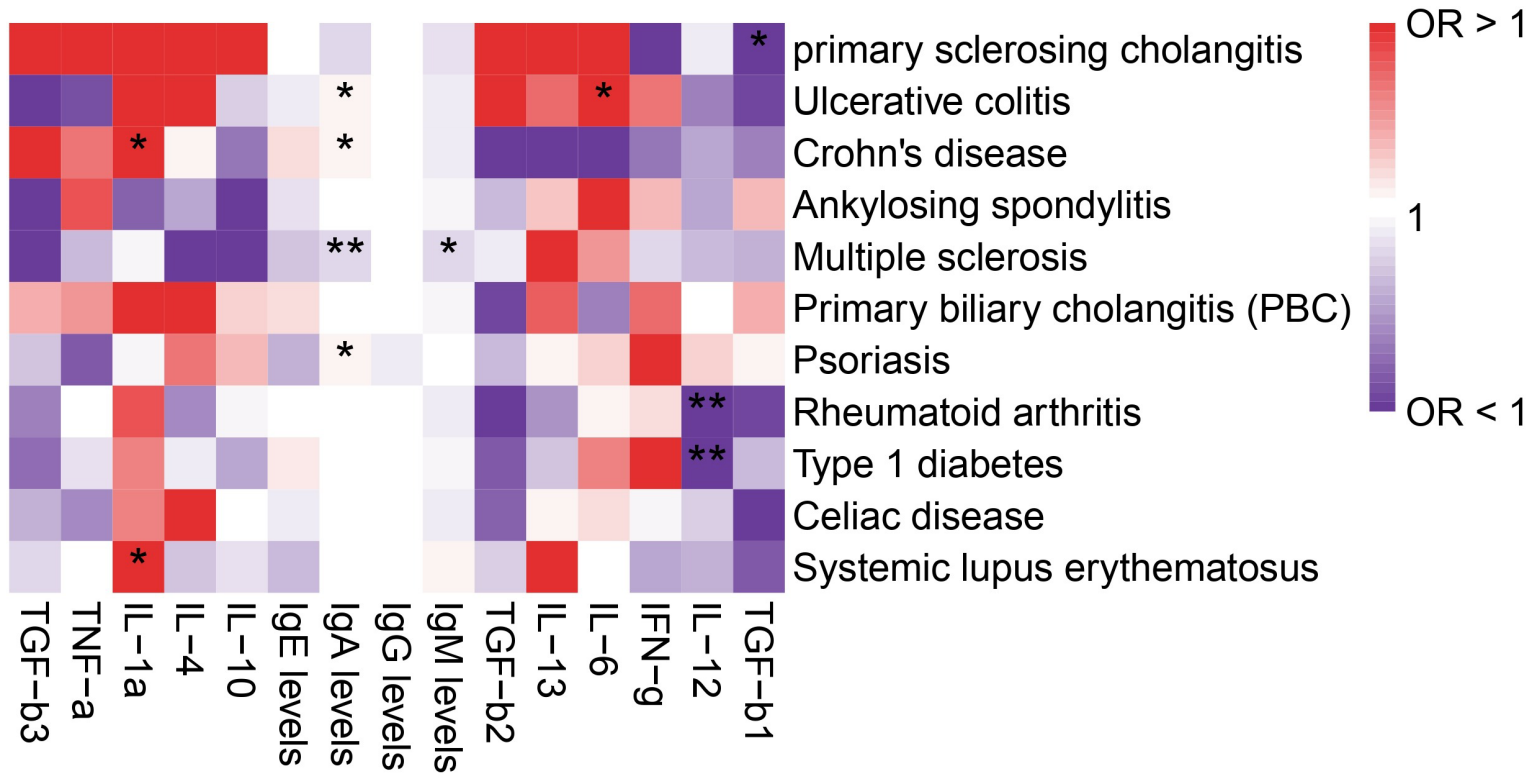
Analysis of the correlation between immune diseases and cytokines. The symbol * indicates a *p*-value less than 0.05, suggesting that the result is statistically significant. The symbol ** represents a *p*-value less than 0.01, indicating a stronger level of statistical significance.

**Figure 4:**
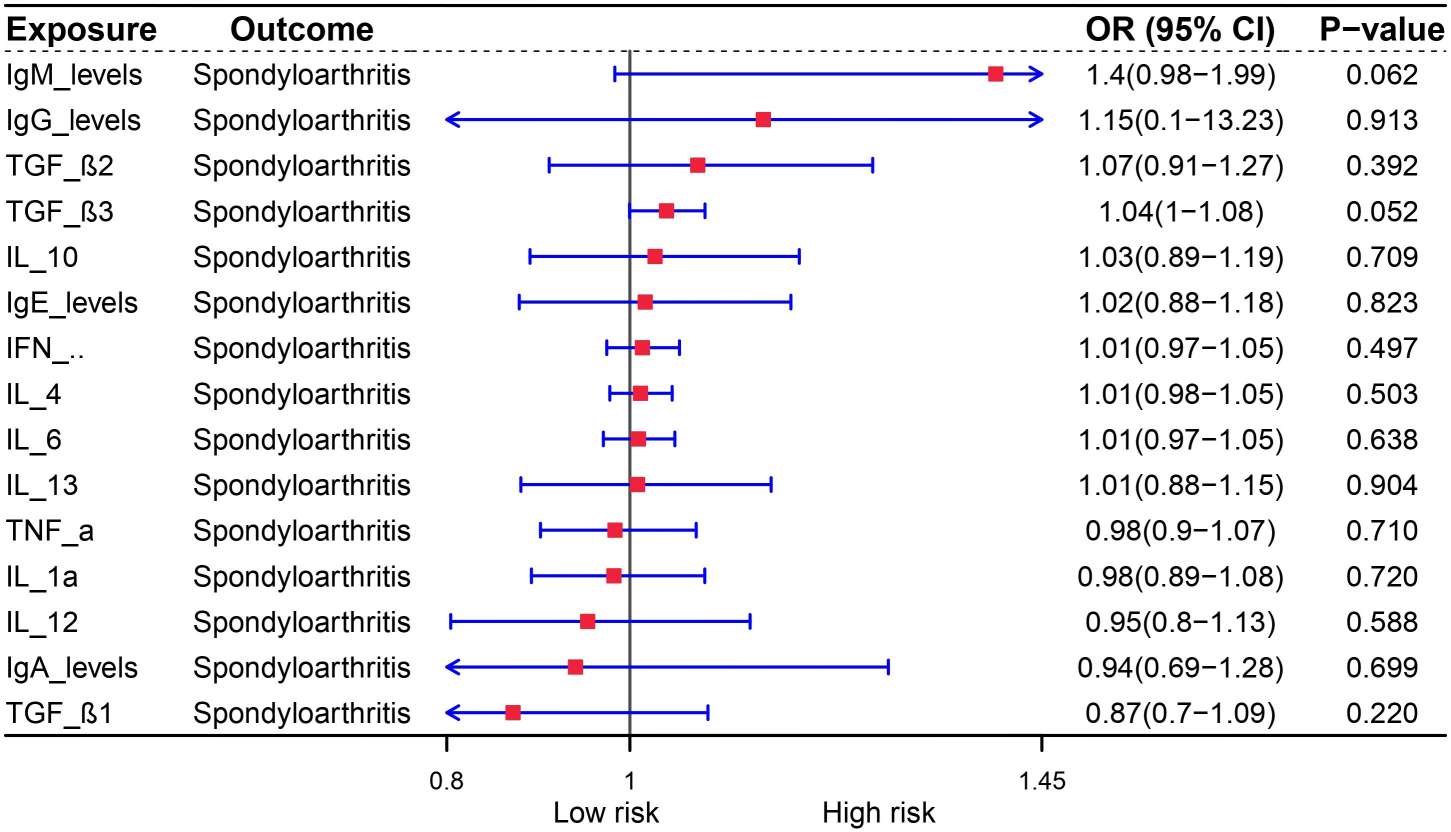
The influence of immune factors on the occurrence of osteoarthritis.

The role of cytokines in autoimmune diseases is of great significance. In order to evaluate the potential of autoimmune diseases to induce osteoarthritis through mediating factors, we incorporated relevant cytokines related to osteoarthritis based on existing literature. Our analysis revealed a strong correlation between cytokines and autoimmune diseases, with IgA levels being specifically linked to Multiple sclerosis, Ulcerative colitis, Psoriasis, and Crohn’s disease. Furthermore, IL-12 was found to be associated with both rheumatoid arthritis and type 1 diabetes (Figure 3). We also investigated the relationship between these cytokines and osteoarthritis, but unfortunately, no mediating factors were identified (Figure 4).

**Figure 5:**
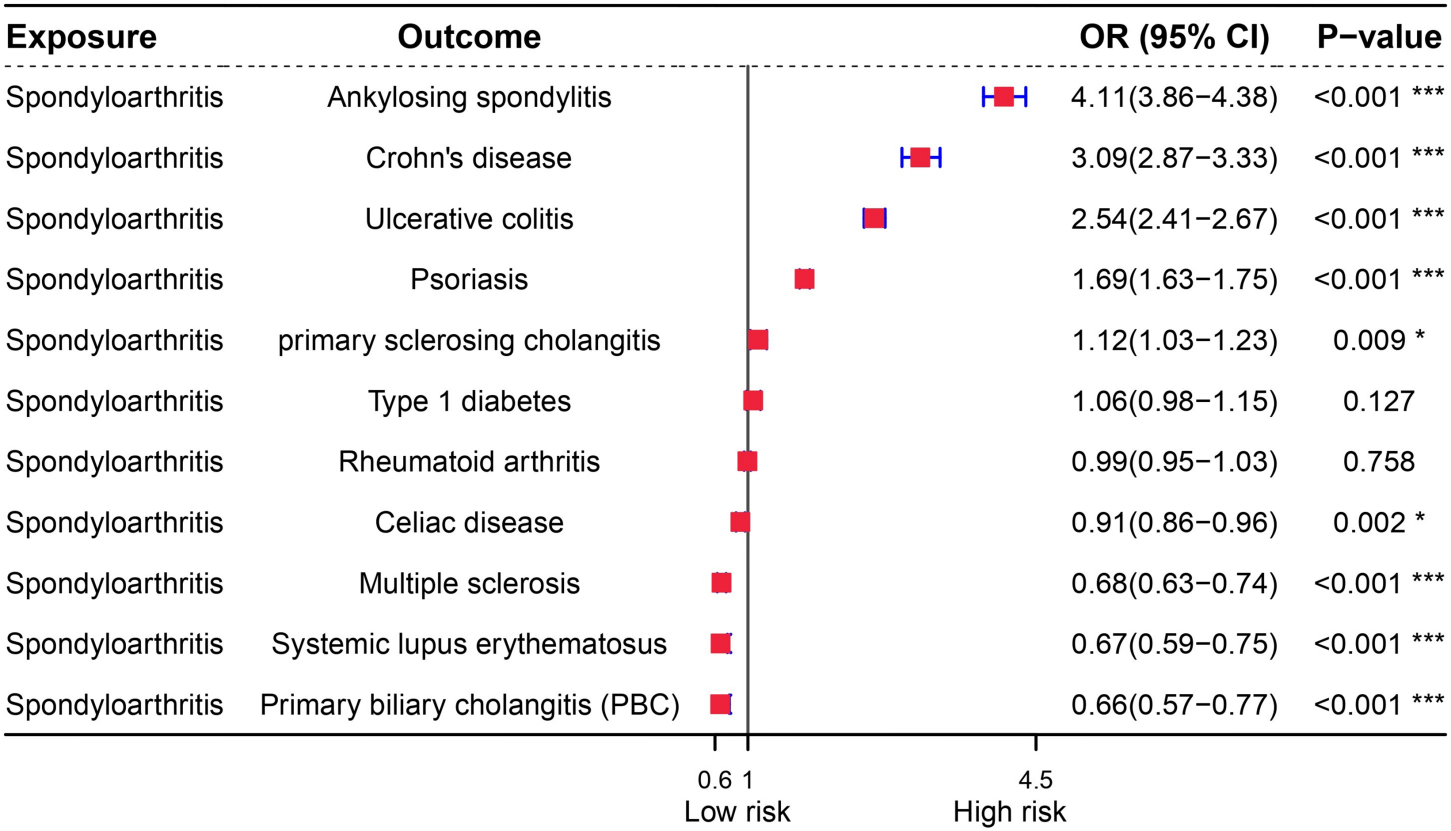
Forest plot of reverse Mendelian randomization analysis for immune diseases and osteoarthritis.

OA is characterized by immunological dysfunction, which has been extensively studied. Previous research has established a link between microbial infections and the development of autoimmune diseases, highlighting the role of immune dysfunction as a triggering factor. In order to further investigate this relationship, we conducted a genetic analysis to determine if there is a causal link between OA and autoimmune diseases. Our reverse MR analysis revealed that the genes associated with OA are also linked to diseases such as Type 1 diabetes and Rheumatoid arthritis. Interestingly, while OA is a risk factor for Ankylosing spondylitis, Crohn’s disease, Ulcerative colitis, and Psoriasis, it is found to be a protective factor for Celiac disease, Multiple sclerosis, Systemic lupus erythematosus, and Primary biliary cholangitis (PBC) (Figure 5).

### Gene expression analysis

**Figure 6:**
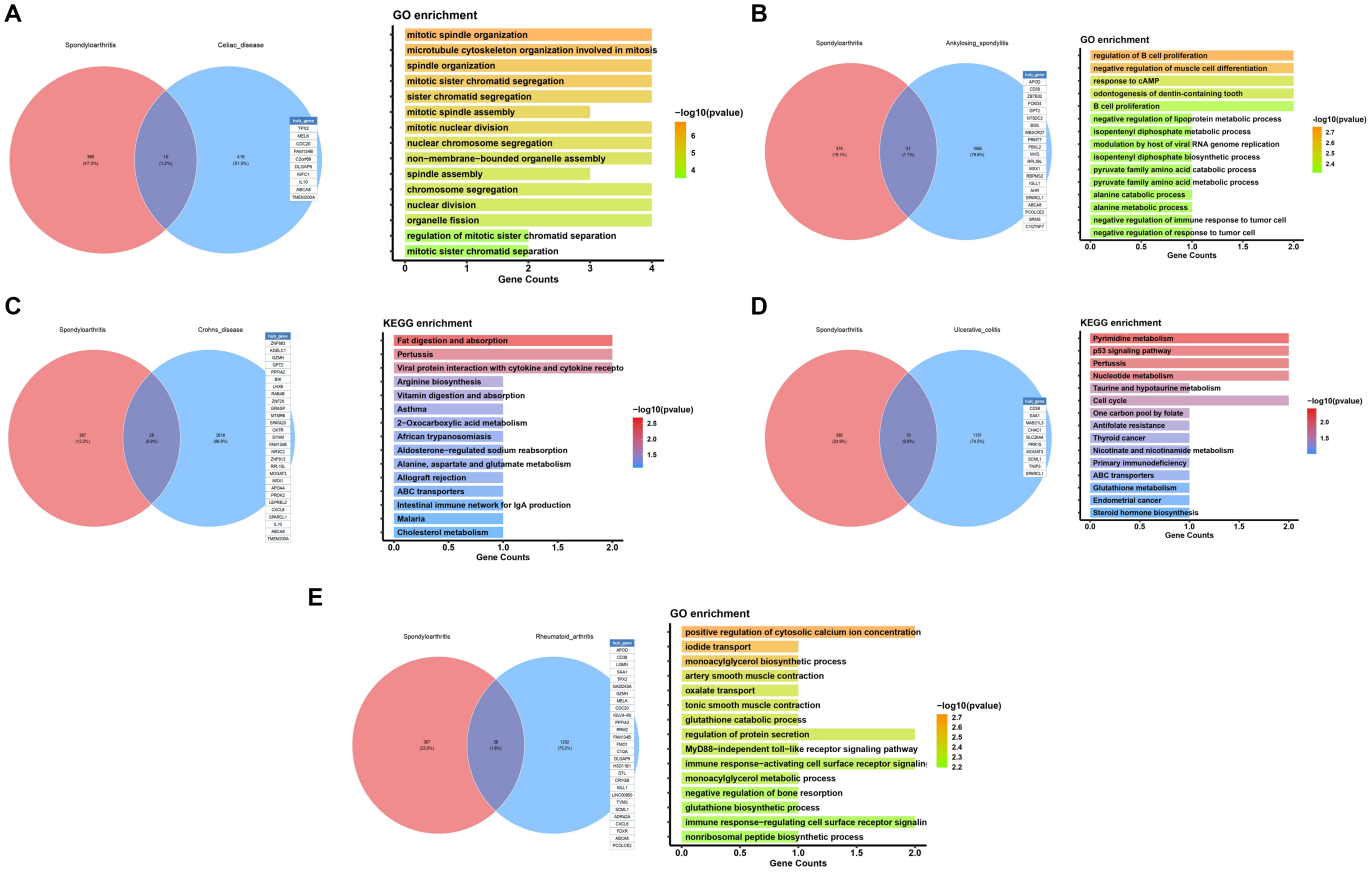
Functional enrichment analysis of potential common mechanisms in osteoarthritis and immune diseases.

In addition to Mendel’s analysis of the genome using randomization, we conducted an examination of common differentially expressed genes between five significant diseases and OA, utilizing publicly available expression profile data. Our analysis revealed a total of 10 differentially expressed genes between OA and Celiac disease, primarily associated with the organization of the mitotic spindle (Figure 6A). Furthermore, we identified 21 genes with differences between OA and Ankylosing spondylitis, mainly related to the regulation of B cell differentiation (Figure 6B). Additionally, there were 28 differentially expressed genes between OA and Celiac disease, with a focus on the digestion and absorption of fat (Figure 6C). We also found 21 genes with differences between OA and Ulcerative colitis, primarily related to Pyrimidine metabolism (Figure 6D). Finally, our analysis identified 28 genes with differences between OA and RA, mainly associated with the positive regulation of cytosolic calcium ion concentration (Figure 6D).

## Discussion

This study conducted a comprehensive investigation into the correlation between autoimmune diseases and osteoarthritis (OA) utilizing both Mendelian randomization (MR) and real-world observational analyses. The findings from the MR study indicate that only genetically predicted rheumatoid arthritis (RA) has a causal relationship with the development of OA (3, 12). While the Mendelian randomization (MR) analysis involving MHC loci SNPs suggests a causal relationship between celiac disease and osteoarthritis (OA), the possibility of pleiotropy resulting in false-positive findings cannot be dismissed. Furthermore, no mediators were identified to clarify the potential association between autoimmune diseases and OA. Importantly, there was no evidence supporting a causal link between these autoimmune conditions and OA. However, reverse MR analyses revealed a possible causal connection between psoriasis and OA.

RA and osteoarthritis (OA) are two of the most common forms of arthritis, affecting millions worldwide. Both conditions lead to significant morbidity and have a profound impact on the quality of life. However, they differ fundamentally in their pathophysiology and clinical manifestations. RA is an autoimmune disease characterized by inflammation of the synovial membrane, leading to joint damage and systemic symptoms. It is associated with autoantibodies such as rheumatoid factor (RF) and anti-citrullinated protein antibodies (ACPA), which are not typically present in OA (13). On the other hand, OA is primarily a degenerative joint disease marked by the breakdown of joint cartilage and underlying bone, typically due to mechanical stress or aging, without the autoimmune component seen in RA (4).Recent studies have highlighted the role of imaging in both diseases, emphasizing its utility in diagnosis and management. Advanced imaging techniques are increasingly used to assess disease progression and response to therapy in clinical trials and practice (5, 14). Additionally, the impact of physical activity and lifestyle modifications on disease outcomes has been recognized, with guidelines suggesting tailored exercise programs for patients with RA and OA to improve function and reduce pain. In our subsequent differential analysis, we found that CD8 is a common risk gene for both diseases. CD38, a multifunctional enzyme involved in calcium signaling and cell adhesion, has been identified as a potential link between RA and OA. Although the specific search results provided do not directly address CD38, it is known from broader scientific literature that CD38 plays a role in the inflammatory processes by regulating the activation and migration of immune cells. This can contribute to the pathogenesis of RA through its effects on immune system dysregulation (15, 16). In OA, while the inflammatory component is less pronounced, CD38 may still contribute to disease progression through pathways involving cellular senescence and tissue remodeling. The differential expression of CD38 in RA and OA could potentially be exploited for diagnostic or therapeutic purposes (17, 18). For instance, targeting CD38 with specific inhibitors could modulate immune responses in RA or could be used to influence the process of cartilage degradation in OA. However, further research is necessary to fully elucidate the role of CD38 in these diseases and to develop targeted therapies that could improve patient outcomes.

Crohn’s disease (CD) and osteoarthritis (OA) are both chronic conditions, traditionally viewed as distinct in their pathophysiology and clinical manifestations. However, recent research has begun to explore potential connections between these diseases, particularly through shared inflammatory pathways and metabolic dysfunctions (19, 20). While the relationship between CD and OA has not been extensively studied, insights can be gleaned from the broader context of inflammatory mechanisms that both conditions may share. For instance, CD is a chronic inflammatory bowel disease primarily affecting the gastrointestinal tract, but systemic inflammation is a well-recognized feature. Similarly, osteoarthritis, traditionally seen as a degenerative joint disease, is now understood to have an inflammatory component, particularly in the synovial membrane. Furthermore, recent research has focused on the role of specific cytokines and inflammatory mediators in both diseases. For example, interleukin-22 (IL-22) has been identified as a significant player in the pathogenesis of CD and is also implicated in the inflammatory processes of OA (18). This commonality suggests shared cytokine-driven inflammatory pathways that could contribute to disease processes in both CD and OA. In our functional enrichment analysis of CD and OA, we found that the two diseases are primarily related to fat digestion and absorption, a metabolism-related pathway. Abnormalities in fat digestion and absorption have been documented in CD, primarily due to intestinal inflammation and malabsorption. Similarly, metabolic syndrome, which includes impaired fat metabolism, has been linked to an increased risk of developing OA. This suggests a potential metabolic link between these two diseases, where dysregulated fat metabolism may contribute to the chronic inflammation observed in both conditions.

Celiac disease and osteoarthritis (OA) are two distinct conditions that have been studied for potential interconnections, particularly at the genetic level. Research into the relationship between these diseases is still in its early stages, with some studies suggesting a possible link through shared genetic markers and inflammatory pathways. One gene of interest in the study of both celiac disease and OA is CDC20. CDC20, or cell division cycle 20, is a regulatory protein that plays a crucial role in the cell cycle by activating the anaphase-promoting complex/cyclosome (APC/C), which is essential for the transition from metaphase to anaphase during cell division (21–28). This gene has been identified as a potential common differential gene in both diseases, suggesting that it might influence the pathophysiology of both celiac disease and OA through its role in cellular processes and inflammation. The impact of CDC20 on these diseases could be multifaceted. In celiac disease, CDC20 might influence the regulation of intestinal epithelial cell proliferation, which is often disrupted in this condition (22). In OA, CDC20 could affect the proliferation of synovial cells or chondrocytes, potentially contributing to the degenerative changes observed in the joint tissues. However, the exact mechanisms through which CDC20 influences these diseases remain unclear and require further investigation.

Ulcerative colitis (UC) and osteoarthritis (OA) are two distinct chronic conditions; UC is an inflammatory bowel disease primarily affecting the colon and rectum, while OA is a degenerative joint disease. Despite their differing areas of impact and underlying causes, recent research suggests a potential link between the inflammatory processes associated with UC and the development or exacerbation of OA. This connection may be partially attributed to shared metabolic pathways, such as pyrimidine metabolism, which plays a crucial role in cellular growth, differentiation, and inflammatory responses. Recent studies have highlighted the significance of pyrimidine metabolism in the pathophysiology of UC. For instance, integrative analyses of transcriptomic and metabonomic profiles have revealed disruptions in pyrimidine metabolism in UC, indicating its involvement in the disease’s inflammatory processes (14, 29). These findings are noteworthy as they suggest that disturbances in pyrimidine metabolism could contribute to the characteristic inflammatory environment of UC, potentially impacting other systems, including the musculoskeletal system. While direct research on the relationship between UC and OA through pyrimidine metabolism is limited, the involvement of this metabolic pathway in inflammatory responses provides a plausible connection. Inflammatory cytokines, which are elevated in UC, have been linked to the development of OA by promoting cartilage degradation and osteophyte formation. Therefore, it is conceivable that the dysregulation of pyrimidine metabolism in UC could exacerbate inflammatory responses, indirectly influencing the development or progression of OA.

Our analysis revealed a correlation between OA and various immune system disorders. This discovery has the potential to guide future clinical treatment processes by identifying mutations in crucial gene loci and targeting them for the treatment of comorbid diseases. This approach may lead to the development of critical drug targets and offer new avenues for treatment.

This study examined the relationship between osteoarthritis and immune diseases through genomics and gene expression analysis. However, there are limitations that should be noted. Due to the variability of GWAS data across populations and regions, the current analysis may not be representative of all populations and further validation with additional samples is necessary. Additionally, while there is some correlation between overlapping genes in different diseases, further specific experiments are required to confirm the accuracy of these findings.

The main objective of this research was to assess the correlation between autoimmune disorders and osteoarthritis using MR and observational studies. Our findings revealed that genes associated with Celiac disease, Crohn’s disease, Ankylosing spondylitis, RA, and Ulcerative colitis were independently linked to the development of OA. Furthermore, we conducted an analysis of potential pathogenic genes between these diseases and OA, offering a novel approach for the simultaneous treatment of multiple conditions.

## Data Availability

The original contributions presented in the study are included in the article/Supplementary material, further inquiries can be directed to the corresponding author.
