# Supplementary material for: Association of autoimmune diseases with the occurrence of osteoarthritis: a gene expression and Mendelian randomization study
Source: Front Med (Lausanne). 2024 Sep 5;11:1435312. doi: 10.3389/fmed.2024.1435312 (PMC11412204; doi:10.3389/fmed.2024.1435312)
Supplement: Supplementary file 1 [file Table_1.docx]

Supplementary table 1 Sample overlap rate analysis table for autoimmune diseases and osteoarthritis

| Trait1 | Trait2 | Case overlap (%) | Bias | Type 1 error rate |
| --- | --- | --- | --- | --- |
| Crohn's disease | Osteoarthritis | 2 | 0.005 | 0.05 |
| Multiple sclerosis | Osteoarthritis | 0.38 | 0.001 | 0.05 |
| Celiac disease | Osteoarthritis | 0.65 | <0.001 | 0.05 |
| Psoriasis | Osteoarthritis | 3.16 | <0.002 | 0.05 |
| Ankylosing spondylitis | Osteoarthritis | 0 |  |  |
| Rheumatoid arthritis | Osteoarthritis | 2.82 | <0.001 | 0.05 |
| Type 1 diabetes | Osteoarthritis | 0.18 | <0.001 | 0.05 |
| Ulcerative colitis | Osteoarthritis | 2.18 | <0.001 | 0.05 |
| Primary biliary cholangitis (PBC) | Osteoarthritis | 0.11 | <0.001 | 0.05 |
| primary sclerosing cholangitis | Osteoarthritis | 0.11 | <0.001 | 0.05 |
| Systemic lupus erythematosus | Osteoarthritis | 0.54 | <0.001 | 0.05 |
